# Topological properties of hydrogen bonds and covalent bonds from charge densities obtained by the maximum entropy method (MEM)

**DOI:** 10.1107/S0108768109026767

**Published:** 2009-08-28

**Authors:** Jeanette Netzel, Sander van Smaalen

**Affiliations:** a Laboratory of Crystallography, University of Bayreuth, D-95440 Bayreuth, Germany

**Keywords:** topological properties, hydrogen bonding, maximum entropy method, charge densities, peptides, amino acids

## Abstract

Charge densities have been determined by the Maximum Entropy Method (MEM) from the high-resolution, low-temperature (*T* ≃ 20 K) X-ray diffraction data of six different crystals of amino acids and peptides. A comparison of dynamic deformation densities of the MEM with static and dynamic deformation densities of multipole models shows that the MEM may lead to a better description of the electron density in hydrogen bonds in cases where the multipole model has been restricted to isotropic displacement parameters and low-order multipoles (*l*_max_ = 1) for the H atoms. Topological properties at bond critical points (BCPs) are found to depend systematically on the bond length, but with different functions for covalent C—C, C—N and C—O bonds, and for hydrogen bonds together with covalent C—H and N—H bonds. Similar dependencies are known for AIM properties derived from static multipole densities. The ratio of potential and kinetic energy densities |*V*(BCP)|/*G*(BCP) is successfully used for a classification of hydrogen bonds according to their distance *d*(H⋯O) between the H atom and the acceptor atom. The classification based on MEM densities coincides with the usual classification of hydrogen bonds as strong, intermediate and weak [Jeffrey (1997) ▶. *An Introduction to Hydrogen Bonding*. Oxford University Press]. MEM and procrystal densities lead to similar values of the densities at the BCPs of hydrogen bonds, but differences are shown to prevail, such that it is found that only the true charge density, represented by MEM densities, the multipole model or some other method can lead to the correct characterization of chemical bonding. Our results do not confirm suggestions in the literature that the promolecule density might be sufficient for a characterization of hydrogen bonds.

## Abstract 2

The maximum-entropy charge densities of six amino acids and peptides reveal systematic dependencies of the properties at bond critical points on bond lengths. MEM densities demonstrate that low-order multipoles (*l*_max_ = 1) and isotropic atomic displacement parameters for H atoms in the multipole model are insufficient for capturing all the features of charge densities in hydrogen bonds.

## 1. Introduction

Inter- and intramolecular hydrogen bonds are important in both molecular and biological chemistry, because they contribute a large part of the interactions responsible for the conformations and functions of many compounds in those fields. Different approaches and methods have been employed to determine geometrical, topological, energetic and functional properties of hydrogen bonds. Besides spectroscopic methods, X-ray diffraction is an important tool for providing answers to structural questions regarding hydrogen bonds. Koch & Popelier (1995 ▶) proposed eight criteria that establish the existence of hydrogen bonds. Geometric, energetic and IR spectroscopic properties were suggested by Jeffrey (1997 ▶), thus allowing a classification of strong, medium and weak hydrogen bonds.

A sophisticated approach to analyze the topological properties of electron densities is provided by Bader’s Atoms in Molecules (AIM) theory (Bader, 1994 ▶). The AIM theory allows the determination of BCPs and their properties, such as the electron density and its Laplacian, leading to the detection of hydrogen bonds in crystal structures as well as providing a quantitative characterization of the type and strengths of these bonds. Analyses according to the AIM theory (Bader, 1994 ▶) of experimental electron densities of amino acids and peptides have been performed by Destro *et al.* (1988 ▶, 2000 ▶), Benabicha *et al.* (2000 ▶), Pichon-Pesme *et al.* (2000 ▶), Wagner & Luger (2001 ▶), Flaig *et al.* (2002 ▶), Scheins *et al.* (2004 ▶), Mebs *et al.* (2006 ▶), Checinska *et al.* (2006 ▶), Rödel *et al.* (2006 ▶) and Kalinowski *et al.* (2007 ▶). Amino acids were studied on the basis of electron densities derived from quantum mechanical calculations by Matta & Bader (2000 ▶, 2002 ▶, 2003 ▶).

Abramov (1997 ▶) introduced a method which uses the densities at BCPs and their Laplacians for calculating the kinetic energy densities at the BCPs. Employment of the local virial theorem (Bader, 1994 ▶) allows the calculation of the potential energy densities at the BCPs. These energy densities provide information on the character of the bond analyzed (Abramov, 1997 ▶; Cremer & Kraka, 1984*a* ▶,*b* ▶). Extensive studies of energy densities and topological properties at the BCPs of hydrogen bonds have been performed by Espinosa *et al.* (1998 ▶, 2002 ▶), Espinosa, Lecomte & Molins (1999 ▶) and Espinosa, Souhassou *et al.* (1999 ▶).

Experimental charge densities are usually based on the multipole model (Hansen & Coppens, 1978 ▶). Alternatively, they can be determined by the maximum entropy method (MEM; Sakata & Sato, 1990 ▶; Hofmann, Kalinowski *et al.*, 2007 ▶; Hofmann, Netzel & van Smaalen, 2007 ▶; Netzel *et al.*, 2008 ▶; Nishibori *et al.*, 2008 ▶). MEM electron densities (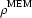) have been successfully used to study disorder in crystal structures. The most prominent application has been the determination of the location of the metal atom in endohedral fullerenes (Takata *et al.*, 1995 ▶). Earlier studies have stressed artifacts in MEM densities, which have magnitudes equal to the deformation densities of chemical bonds, and thus would prohibit the use of the MEM in charge-density studies (Jauch & Palmer, 1993 ▶; Jauch, 1994 ▶; de Vries *et al.*, 1996 ▶; Takata & Sakata, 1996 ▶; Roversi *et al.*, 1998 ▶). These problems have been overcome by a combination of extensions to the MEM, including the use of a procrystal prior density (de Vries *et al.*, 1996 ▶), the use of static weights in the 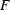 constraint (de Vries *et al.*, 1994 ▶), the use of prior-derived 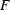 constraints (Palatinus & van Smaalen, 2005 ▶) and the definition of a criterion of convergence for the MEM iterations, which is based on difference-Fourier maps (Hofmann, Netzel & van Smaalen, 2007 ▶). The MEM has the potential to become the method of choice in accurate charge-density studies on proteins (Hofmann, Kalinowski *et al.*, 2007 ▶; Nishibori *et al.*, 2008 ▶), because the MEM (unlike multipole refinements) does not suffer from correlations between parameters.

The present work reports the analysis of MEM electron densities of several amino acids and peptides. The study includes the analysis of geometrical, topological and energetic properties of all 52 hydrogen bonds that have been identified in these compounds. The quantitative analysis is supplemented by a descriptive analysis of electron densities in the regions of the hydrogen bonds. Since the role of a promolecule (procrystal) has been discussed as being important for the extraction of information of bonding (Spackman, 1999 ▶; Downs *et al.*, 2002 ▶), the contribution of the prior density to properties of chemical bonds is discussed. The systematic dependence of properties of hydrogen bonds on the distance between the H atom and acceptor atom is supplemented by an analysis of the properties of covalent bonds with respect to the bond distance.

## 2. Computational details

### 2.1. MEM calculations

Single-crystal X-ray diffraction data of l-alanine (Ala; Destro *et al.*, 1988 ▶), an l-phenylalanine formic acid complex (Phe; Mebs *et al.*, 2006 ▶), l-alanyl-l-tyrosyl-l-alanine (Ala–Tyr–Ala) with water as the solvent and Ala–Tyr–Ala with ethanol as the solvent (Checinska *et al.*, 2006 ▶) were kindly provided by Destro or by Luger and co-workers, who have already reported multipole refinements against these data (Table 1 ▶). We have used these data to perform refinements of the independent spherical atom model (ISAM) with the computer program *JANA*2000 (Petříček *et al.*, 2000 ▶). The coordinates and atomic displacement parameters (ADPs) obtained by the multipole refinements were used as the starting model for the ISAM refinement. H atoms were fixed at distances known from neutron diffraction (Wilson, 1995 ▶; Mebs *et al.*, 2006 ▶; Baur, 1972 ▶; Ohtomo & Arakawa, 1995 ▶). A riding model of 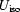
(H) = 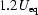
(N, C) and 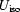
(H) = 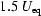
(O) was employed to calculate the ADPs of H atoms. For all three water molecules of the asymmetric unit of Ala–Tyr–Ala with water, the H—O—H angle was restrained to 104.5°. The coordinates of H2b of Phe, of H15, H61, H62, H71, H72, H81 and H82 of Ala–Tyr–Ala with water, and of H15 and H16 of Ala–Tyr–Ala with ethanol were independently refined subject to constraints on the *X*—H distances towards values known from neutron diffraction, because a reasonable geometrical restraint was not available for these H atoms. Coordinates of all other H atoms were obtained by attachment to their neighbor atoms with tetrahedral or trigonal angle restraints according to their chemical meaning. Agreement indices for ISAM refinements are given in Table 1 ▶.

According to a procedure by Bagautdinov *et al.* (1998 ▶), the ISAM refinement was employed to obtain phased and scaled observed structure factors corrected for anomalous scattering, which were used for the MEM calculations. The coordinates and ADPs of the ISAM were used to compute the procrystal electron density [prior density or prior, 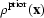] with the computer program *PRIOR* (van Smaalen *et al.*, 2003 ▶). The prior was calculated on a grid over the unit cell and used as the reference density in the MEM calculations. Equal grids were chosen for the prior and MEM densities, such that the pixel size did not exceed 0.1 × 0.1 × 0.1 Å^3^ (Table 1 ▶).

The MEM is based on the principle that the most probable density 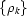 is that which simultaneously fits the diffraction data and maximizes the informational entropy 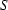, with 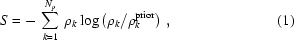
where 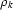 = 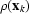 are the values of the electron density on a grid over the unit cell of 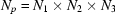 points. 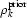 = 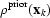 are the corresponding values of the prior.

Diffraction data are taken into account by the method of undetermined Lagrange multipliers employing the 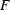 constraint 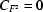 with (Sakata & Sato, 1990 ▶; Hofmann, Netzel & van Smaalen, 2007 ▶)
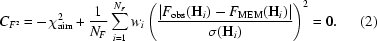

               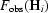 is the phased observed structure factor of the Bragg reflection with scattering vector 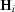 and standard uncertainty (s.u.) 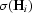. 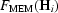 is obtained by discrete Fourier transform of the electron density 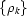. The summation extends over all observed reflections 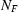. Static weights 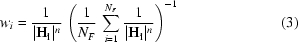
with 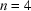 have been chosen according to de Vries *et al.* (1994 ▶). Our earlier studies have confirmed that weights H4 represent the optimal choice of weights (Hofmann, Netzel & van Smaalen, 2007 ▶; Netzel *et al.*, 2008 ▶). The summation of (2) has been extended towards all reflections up to 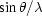
 = 2.5 Å^−1^ according to the method of prior-derived 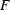 constraints (PDC; Palatinus & van Smaalen, 2005 ▶). Since the PDC extends the 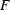 constraint using terms involving the calculated structure factors of the prior, its use is recommended only if the experimental data are available up to a certain minimum resolution, *e.g.* up to at least 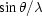
 > 0.9 Å^−1^ (Palatinus & van Smaalen, 2005 ▶). This condition is fulfilled for all the datasets considered in this article.

MEM calculations have been performed with the computer program *BayMEM* (van Smaalen *et al.*, 2003 ▶), employing the Cambridge maximum entropy algorithm (Gull, 1989 ▶; Gull & Skilling, 1999 ▶). Convergence of the iterations is considered to be reached once 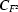 has dropped below zero [see (2)], and it thus depends on the value of 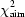. Following procedures introduced earlier (Netzel *et al.*, 2008 ▶; Hofmann, Netzel & van Smaalen, 2007 ▶), an optimal value for 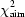 was determined for each dataset by inspection of difference-Fourier maps and dynamic deformation maps 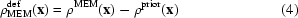
for several values of 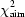 (Table 1 ▶). Details of the MEM calculations of 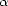-glycine and trialanine have been described elsewhere (Netzel *et al.*, 2008 ▶; Hofmann, Netzel & van Smaalen, 2007 ▶).

An optimal value for 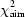 is necessary to obtain accurate and reliable electron-density maps by the MEM (Hofmann, Kalinowski *et al.*, 2007 ▶). In theory (Skilling, 1989 ▶; Gull, 1989 ▶), one would only expect values of 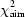
 < 1, but values of 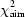
 > 1 can appear if the standard uncertainties of measured reflection intensities have been estimated to be smaller than their true values. The standard uncertainties in turn are related to the goodness of fit (GoF) of the refinements, with 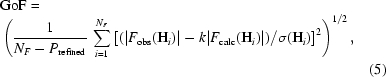
where 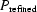 is the number of refined parameters, 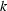 is the scale factor and 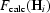 are the calculated structure factors of the model. For underestimated standard uncertainties, the value of GoF will be larger than that at convergence of the refinement.

For datasets with multipole refinements resulting in a GoF ≃ 1, we obtained 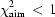, and for datasets with refinements resulting in a GoF close to two, values of 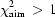 were obtained (Table 1 ▶). This indicates that the stopping criterion for the MEM calculation depends on the scale of the standard uncertainties of the intensities. Thus, the accuracy of the standard uncertainties can be estimated from consideration of the value of 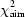 as determined in the MEM procedure. Values of 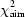 smaller than one indicate that the standard uncertainties are estimated close to their true values, whereas values of 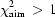 indicate that the standard uncertainties have been underestimated.

### 2.2. Analysis of the MEM density

**Figure 1:**
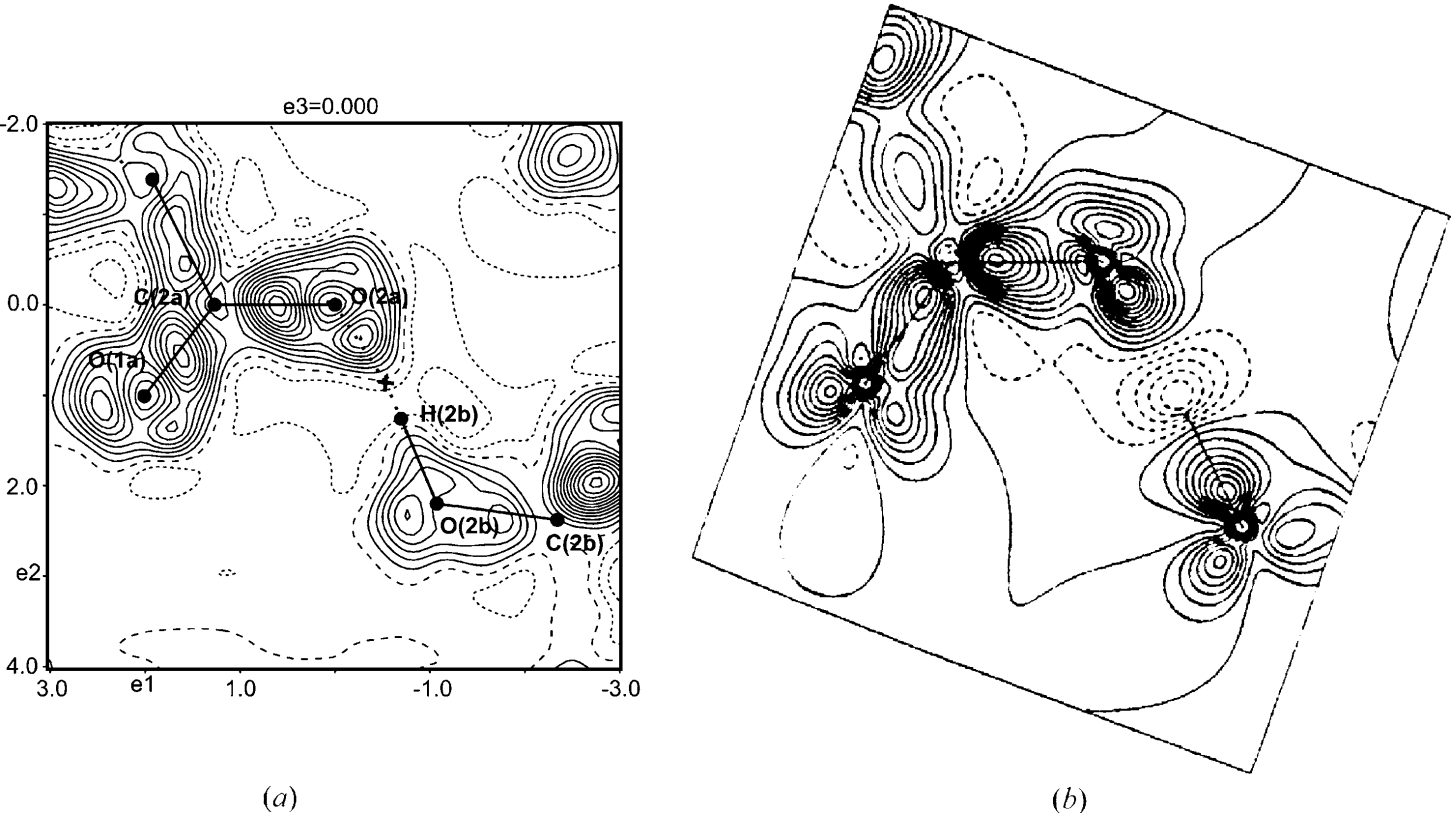
Sections of deformation maps of the l-phenylalanine formic acid complex, containing the atoms O2a—C2a—O1a and showing the hydrogen bond O2b—H2b⋯O2a. (*a*) Section of area 6 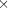 6 Å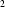 of the dynamic deformation density of the MEM [see (4)]. Contour intervals: 0.05 e Å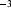. Solid lines indicate positive contours, dotted lines negative contours and dashed lines represent the zero contour. 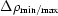 = −0.15/0.61 e Å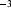. The cross indicates the BCP of the hydrogen bond, with *d*(H⋯O) = 1.45 Å, 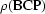 = 0.599 e Å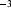 and 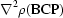 = 0.32 e Å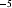. (*b*) Static deformation density of the multipole model (reprinted with permission from Mebs *et al.*, 2006 ▶). Contour intervals: 0.10 e Å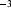.

The difference between the ISAM and the aspherical electron distribution obtained by the MEM has been analysed by inspection of dynamic deformation maps [see (4)]. In particular, sections of 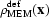 containing selected atoms allow the visualization of the difference densities in hydrogen bonds (Fig. 1 ▶).

Electron-density maps have been analysed according to Bader’s AIM theory (Bader, 1994 ▶) with the module *EDMA* of the program *BayMEM* (van Smaalen *et al.*, 2003 ▶). For each density map, EDMA provides the positions and values of local maxima of the density, the atomic basins, the atomic charges and the positions of BCPs together with their densities 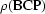, their principal curvatures 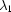, 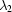 and 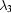 (eigenvalues of the Hessian matrix), and their Laplacians 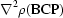. Both the prior and the MEM densities have been analysed in the same way. Covalent bonds have been identified by BCPs with values of 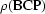 larger than ∼ 1.0 e Å^−3^. A BCP with a smaller value of 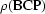 in the region of a potential donor–acceptor pair for hydrogen bonds was used to establish the existence of a hydrogen bond. The positions of BCPs of covalent and hydrogen bonds in MEM densities match BCPs in electron densities obtained from the multipole model.

The kinetic, potential and total energy densities at BCPs have been obtained from prior and MEM densities according to a procedure proposed by Abramov (1997 ▶). The kinetic energy density 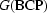 at a BCP is given by 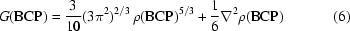
with 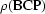 and 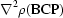 in atomic units. Employing the local virial theorem (Bader, 1994 ▶), the potential energy density 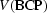 at a BCP is 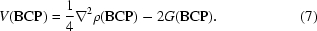
The total energy density 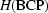 at a BCP then is defined as 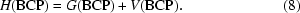
Note that (6) and (7) have been derived for static electron densities, *i.e.* within the Born–Oppenheimer approximation. We apply these relations to dynamic densities as obtained by the prior and the MEM. While systematic dependencies of, for example, 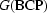 on *d*(H⋯O) are found (§3.3), the interpretation of these quantities as kinetic and potential energy densities needs to be established or correction factors need to be found (see the discussion in §3.3). This is beyond the scope of the present manuscript.

Systematic dependencies on bond lengths have been established for various topological and energetic properties at BCPs of covalent C—C, C—N, C—O, C—H and N—H bonds and of hydrogen bonds. Since some H atoms do not constitute atomic maxima, the corresponding coordinates of H atoms from the ISAM were employed to calculate the distance *d*(H⋯O).

## 3. Results and discussion

### 3.1. Electron densities in hydrogen bonds

The dynamic deformation map of the MEM [see (4)] and the static deformation map of the multipole model have similar appearances for the l-phenylalanine formic acid complex (Fig. 1 ▶). Distinct features, like lone pairs of O atoms and an accumulation of electron density in regions of covalent bonding, are uncovered by both the MEM and the multipole model. However, the hydrogen bond appears differently in these two densities: along the bond path of the hydrogen bond, the MEM leads to a positive difference density (Fig. 1 ▶ *a*), whereas the multipole method exhibits a negative deformation density in this region (Fig. 1 ▶ *b*). These observations are consistent with those on trialanine and 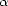-glycine (Hofmann, Netzel & van Smaalen, 2007 ▶; Netzel *et al.*, 2008 ▶). They can be interpreted as being due to:

(i) the differences between dynamic and static densities,
(ii) features of the MEM, *e.g.* its tendency to produce densities as flat as possible, and
(iii) the known inflexibility of the multipole model in describing densities at positions remote from atomic maxima and, especially, the limitations of the multipole model in describing densities around H atoms if the latter have been modeled by isotropic ADPs and low-order multipoles (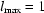) (Volkov *et al.*, 2000 ▶, 2001 ▶; Volkov & Coppens, 2001 ▶; Madsen *et al.*, 2004 ▶; Koritsanszky, 2006 ▶).

**Figure 2:**
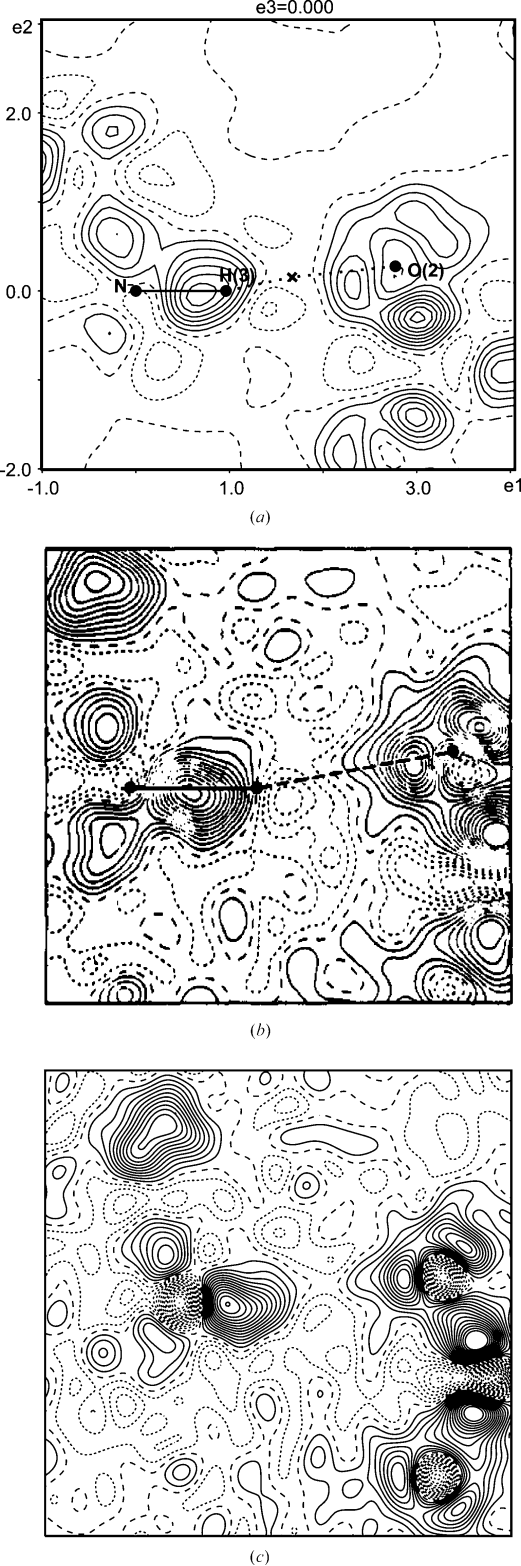
Sections of deformation densities containing the atoms N—H3—O2 and showing the hydrogen bond N—H3⋯O2 of l-alanine. (*a*) Section of area 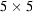
 Å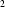 through the dynamic deformation density of the MEM [see (4)]. 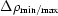 = −0.13/0.42 e Å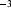. The cross indicates the BCP of the hydrogen bond with *d*(H⋯O) = 1.76 Å, 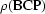 = 0.326 e Å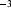 and 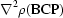 = −1.46 e Å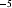. (*b*) Section of 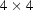
 Å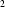 through the experimental dynamic deformation density (reprinted with permission from Destro *et al.*, 1988 ▶). (*c*) Section of 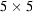
 Å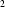 through the experimental dynamic deformation density with phases from the extended multipole model (Destro *et al.*, 2008 ▶; Destro & Lo Presti, 2008 ▶). Contour intervals: 0.05 e Å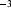; contour lines as defined in Fig. 1 ▶.

Further support for this interpretation comes from the comparison of the dynamic deformation density of the MEM [see (4)] with an experimental dynamic deformation map that has been computed as the difference-Fourier map of 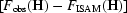, whereby phases for 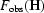 have been obtained from a multipole model (Destro *et al.*, 1988 ▶). The deformation density 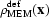 [see (4)] along the bond path of the N—H⋯O hydrogen bond in l-alanine exhibits similar features as 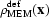 of the O—H⋯O hydrogen bond in l-phenylalanine (Figs. 1 ▶ *a* and 2 ▶ *a*). The experimental difference-Fourier map with phases from a standard multipole model exhibits a deformation density of N—H that is less protruded towards oxygen than 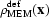, while the minimum density along the H⋯O bond path is approximately 0.1 e Å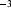 lower than in 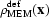 (Fig. 2 ▶ *b*). Phases of an extended multipole model (anisotropic ADPs and multipole parameters up to quadrupole terms for hydrogen) then lead to a dynamic difference-Fourier map that is closer to 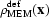 (Fig. 2 ▶ *c*; Destro *et al.*, 2008 ▶).

It has been noticed that anisotropic ADPs and higher-order multipole terms of H atoms are important for a proper description of the electron density around H atoms (Madsen *et al.*, 2004 ▶; Roversi & Destro, 2004 ▶; Whitten *et al.*, 2006 ▶). However, a refinement of these parameters is not possible for systems substantially larger than simple amino acids, owing to the problem of dependent parameters in the multipole model. Usually, the treatment of H atoms does not go beyond isotropic ADPs (Munshi *et al.*, 2008 ▶; Benabicha *et al.*, 2000 ▶; Pichon-Pesme *et al.*, 2000 ▶; Kalinowski *et al.*, 2007 ▶; Wagner & Luger, 2001 ▶; Lyssenko *et al.*, 2005 ▶) and dipolar terms within the multipole model (Grabowsky *et al.*, 2007 ▶; Wagner *et al.*, 2004 ▶; Checinska *et al.*, 2006 ▶; Dominiak *et al.*, 2006 ▶). The extended multipole model will thus remain an exceptional case, to be encountered for crystals of small molecules only. On the other hand, the MEM is applicable to both small and large systems and it leads to a proper description of the deformation density with phases from the ISAM.

**Figure 3:**
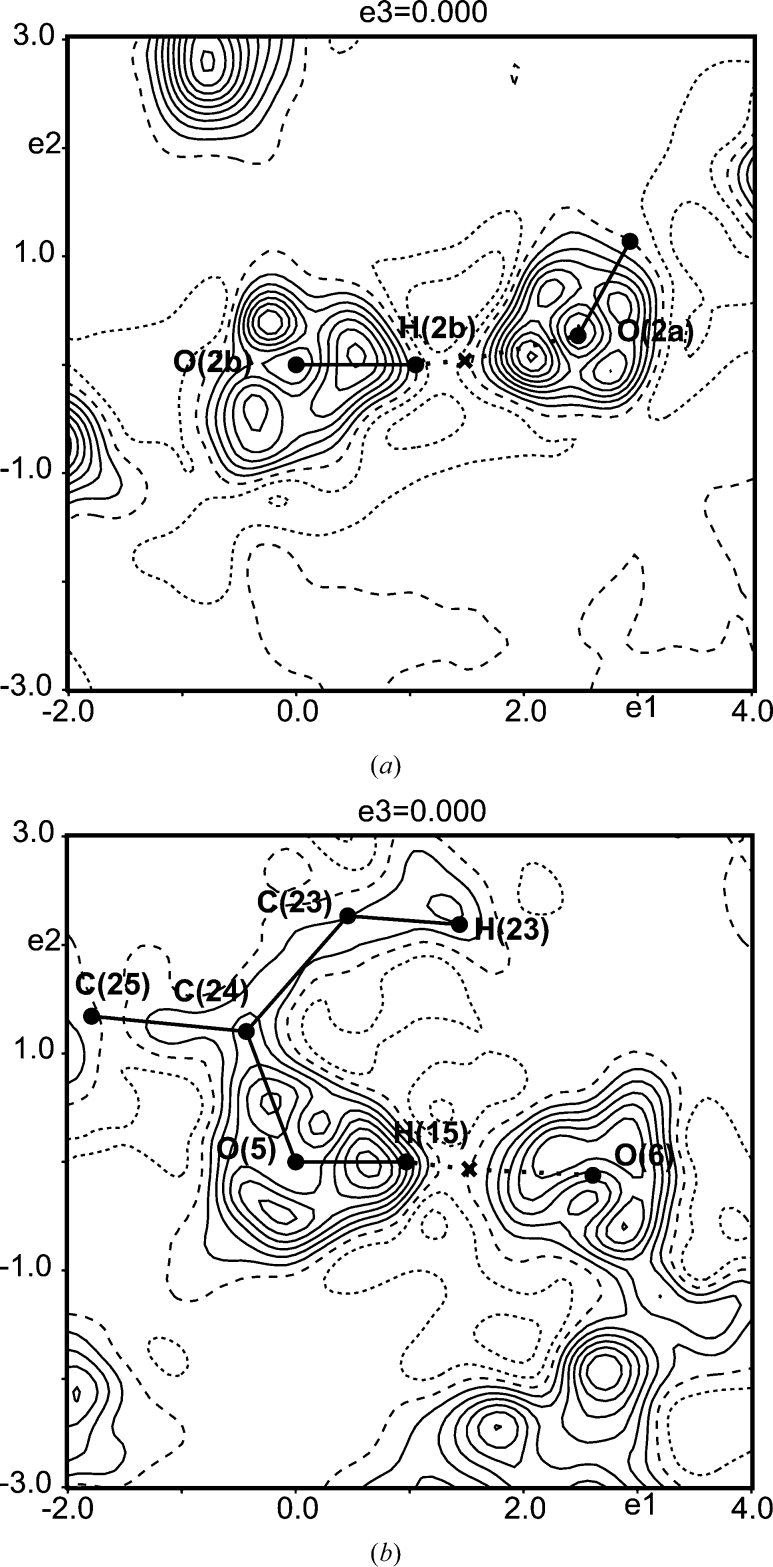
Sections of area 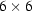
 Å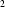 of dynamic deformation densities [see (4)] showing hydrogen bonds of the type O—H⋯O. (*a*) The plane containing the atoms O2b—H2b—O2a of the l-phenylalanine formic acid complex. 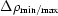 = −0.15/0.46 e Å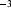. This is a different plane containing the same hydrogen bond as displayed in Fig. 1 ▶(*a*). (*b*) The plane containing the atoms O5—H15—O6 of Ala–Tyr–Ala with ethanol. 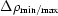 = −0.12/0.37 e Å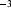. Properties of the hydrogen bond O5—H15⋯O6: *d*(H⋯O) = 1.67 Å, 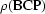 = 0.435 e Å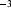 and 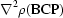 = 2.05 e Å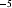. Crosses indicate BCPs; contour interval: 0.05 e Å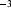; contour lines as defined in Fig. 1 ▶.

**Figure 4:**
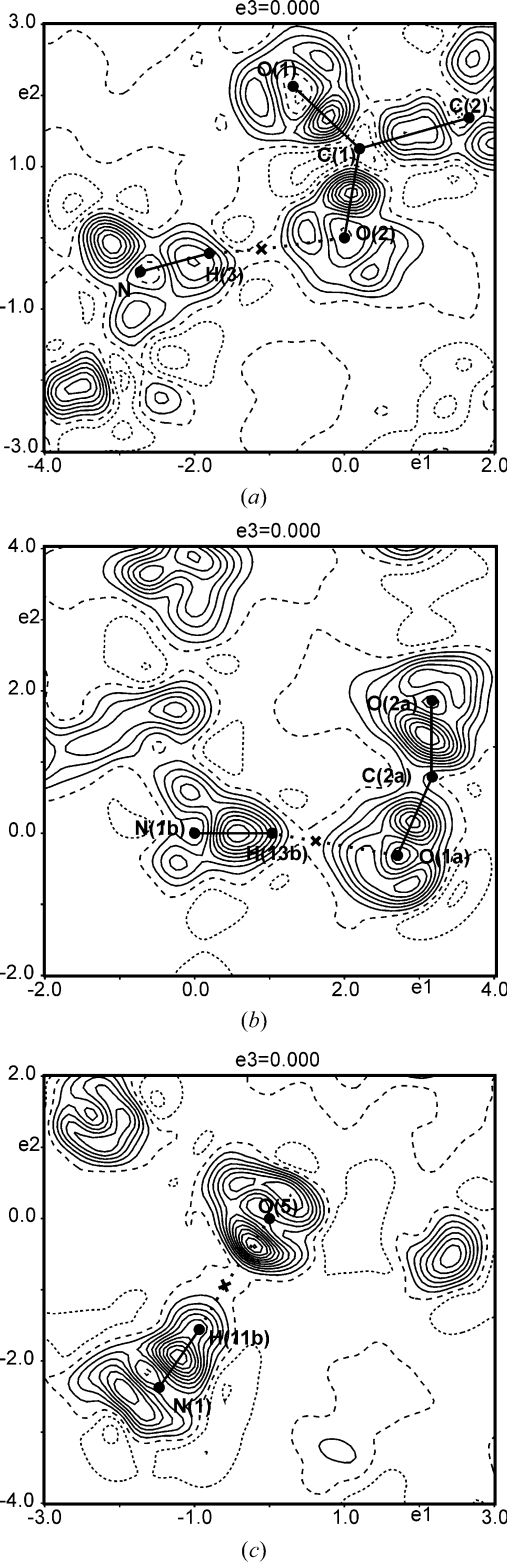
Sections of area 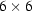
 Å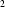 of dynamic deformation densities [see (4)] showing hydrogen bonds of the type N—H⋯O. (*a*) The plane containing the points O2—BCP—H3 of l-alanine. 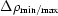 = −0.13/0.43 e Å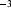. This is a different plane containing the same hydrogen bond as displayed in Fig. 2 ▶(*a*). (*b*) The plane containing the atoms N1b—H13b—O1a of the l-phenylalanine formic acid complex. 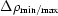 = −0.11/0.50 e Å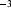. Properties of the hydrogen bond N1b—H13b⋯O1a are: *d*(H⋯O) = 1.71 Å, 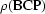 = 0.402 e Å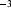 and 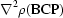 = 2.82 e Å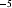. (*c*) The plane containing the atoms O5—H11b—N1 of Ala–Tyr–Ala with water. 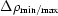 = −0.12/0.56 e Å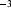. Properties of the hydrogen bond N1—H11b⋯O5 are: *d*(H⋯O) = 1.76 Å, 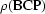 = 0.364 e Å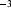 and 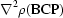 = 0.57 e Å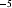. Crosses indicate BCPs, contour intervals: 0.05 e Å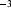, contour lines as defined in Fig. 1 ▶.

**Figure 5:**
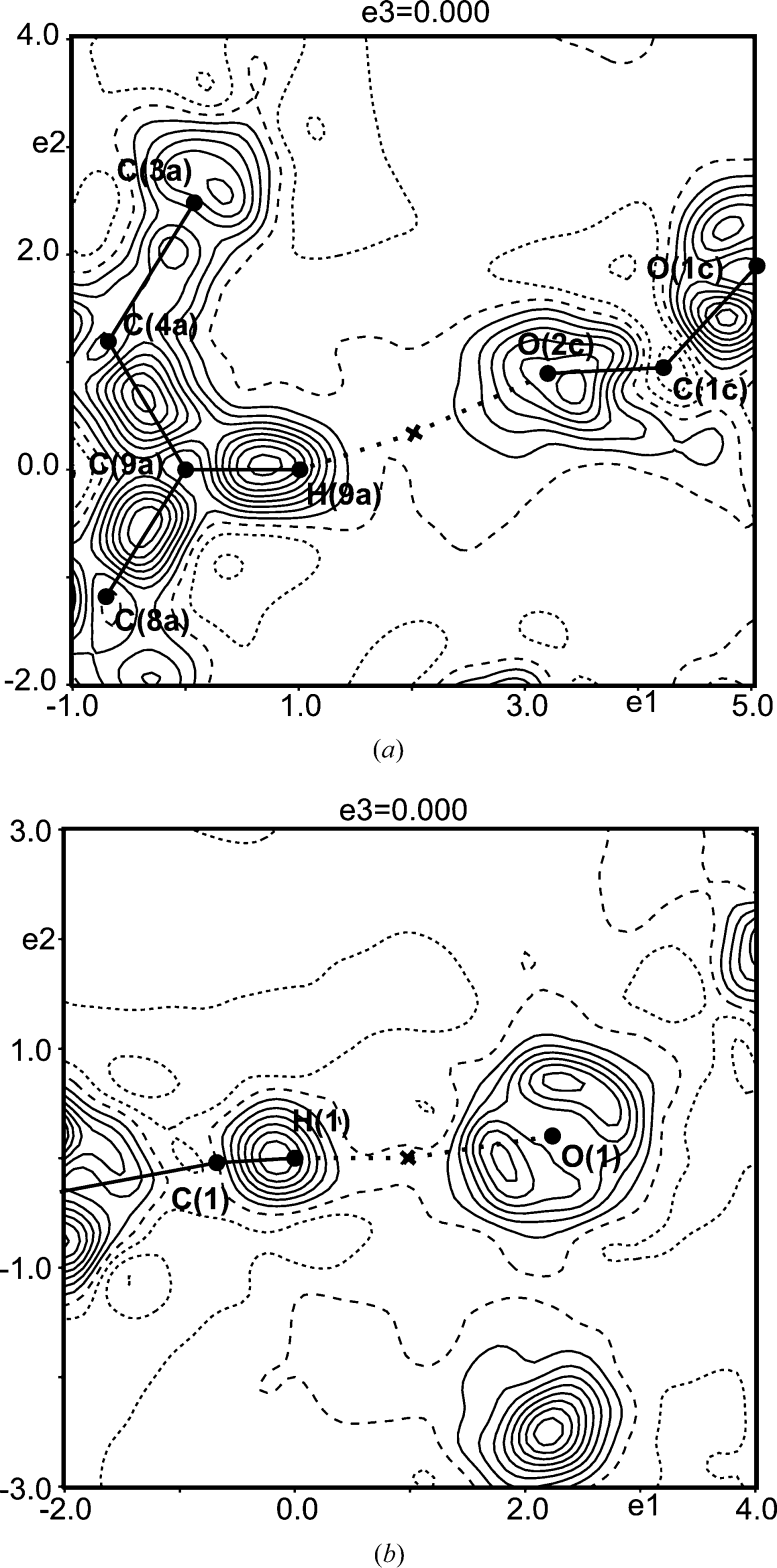
Sections of area 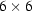
 Å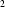 of dynamic deformation densities [see (4)], showing hydrogen bonds of the type C—H⋯O. (*a*) The plane containing the atoms C9a—H9a—O2c of the l-phenylalanine formic acid complex. 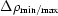 = −0.13/0.42 e Å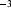. Properties of the hydrogen bond C9a—H9a⋯O2c are: *d*(H⋯O) = 2.36 Å, 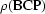 = 0.111 e Å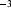 and 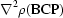 = 0.91 e Å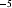. (*b*) The plane containing the points H1—BCP—O1 of Ala–Tyr–Ala with ethanol. 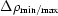 = −0.15/0.43 e Å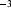. Properties of the hydrogen bond C1—H1⋯O1 are: *d*(H⋯O) = 2.19 Å, 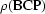 = 0.150 e Å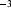 and 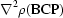 = −0.25 e Å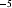. Crosses indicate BCPs, contour intervals at 0.05 e Å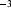, contour lines as defined in Fig. 1 ▶.

Positive dynamic difference densities around the BCPs between the H atom and the acceptor atom turn out to be a feature of all three types of hydrogen bonds studied in the present work. For hydrogen bonds of the type O—H⋯O and N—H⋯O, this feature is very pronounced (Figs. 1–4 ▶ ▶ ▶ ▶), whereas this behaviour is less pronounced in hydrogen bonds of the type C—H⋯O (Fig. 5 ▶). Since hydrogen bonds of the latter type can be considered as very weak or even as non-conventional hydrogen bonds (Marechal, 2007 ▶), the present results confirm that only stronger hydrogen bonds have a large potential to draw electrons into the BCP, resulting in an accumulation of charge between the H atom and the acceptor atom.

### 3.2. Topological properties of hydrogen bonds

**Figure 6:**
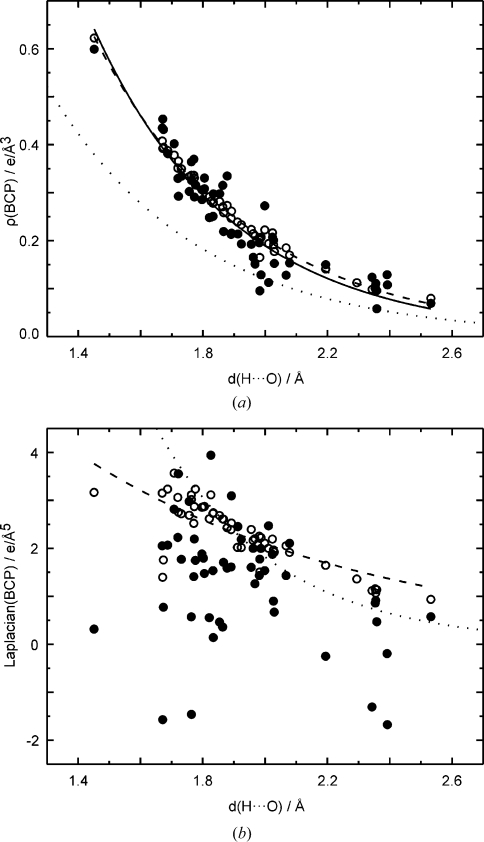
Topological properties of hydrogen bonds from MEM (filled circles) and prior (open circles) densities depending on the distance *d*(H⋯O). (*a*) 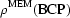 and (*b*) 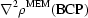. The solid line represents the fit to the MEM values given by 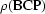 = 16.14 (3.76) exp[−2.22 (13) *d*(H⋯O)]. Dashed lines are fits to PRIOR values, with 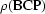 = 12.27 (68) exp[−2.05 (3) *d*(H⋯O)] and 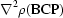 = 17.77 (4.35) exp[−1.07 (13) *d*(H⋯O)]. Dotted lines are functions derived by Espinosa, Souhassou, Lachekar & Lecomte (1999 ▶) from fits to topological properties of multipole densities, with 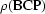 = 8 (4) exp[−2.1 (3) *d*(H⋯O)] and 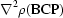 = 330 (180) exp[−2.6 (3) *d*(H⋯O)].

Densities at the BCPs of hydrogen bonds of both MEM and prior densities depend exponentially on the distance *d*(H⋯O) (Fig. 6 ▶ *a*). For the prior this dependence is almost perfect, while values of 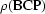 derived from MEM densities exhibit a larger scatter about the exponential curve. Contributions to this scatter come from the properties of the MEM that it will have fitted part of the noise in the data and that it suffers from series termination effects owing to the incompleteness of the data. Part of the scatter of values will be a real property caused by different bonding properties of hydrogen bonds of similar lengths. Furthermore, part of the scatter of values will be due to differences in thermal motion of atoms involved in similar hydrogen bonds, thus leading to differences in dynamic densities even if the static density would be similar.

A quantum mechanical theory does not exist which would demand an exponential relationship between 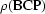 and *d*(H⋯O). Deviations from an average exponential relation can thus be caused by variations of the properties of the bonds, *e.g.* as caused by variations of their environments. It is noted that Espinosa, Souhassou, Lachekar & Lecomte (1999 ▶) have established an exponential relationship between 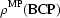 of hydrogen bonds and *d*(H⋯O) for static multipole densities, albeit with different values of the parameters in the exponential function than have presently been determined for MEM and prior densities (Fig. 6 ▶). The values of 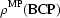 also show a substantial scatter about the proposed exponential dependence on the distance *d*(H⋯O) (Espinosa, Souhassou, Lachekar & Lecomte, 1999 ▶).

The dynamic densities at the BCPs of hydrogen bonds in the prior and the MEM densities are in general larger than the corresponding values of the static multipole density. In view of the discussion in §3.1, we believe this to be a real effect that is caused by the dynamic *versus* static character of the densities and by the inflexibility of the multipole model in the region of hydrogen bonds due to the limited number of poles (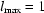) that have been used for H atoms (Volkov *et al.*, 2000 ▶, 2001 ▶; Volkov & Coppens, 2001 ▶; Madsen *et al.*, 2004 ▶; Koritsanszky, 2006 ▶).

**Figure 7:**
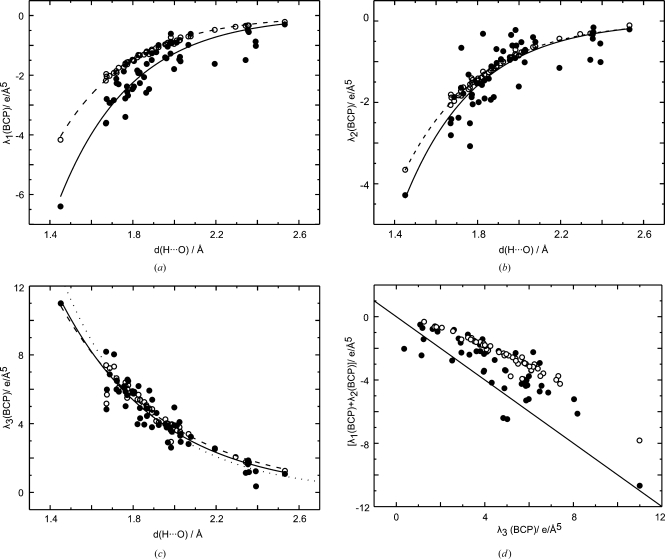
Curvatures at the BCPs of hydrogen bonds for MEM (filled circles) and prior (open circles) densities. (*a*) 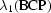, (*b*) 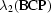 and (*c*) 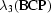 as a function of the distance *d*(H⋯O). (*d*) 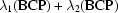 *versus* 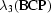. Solid lines represent fits to MEM values with 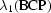 = −378.51 (117.17) exp[−2.85 (18) *d*(H⋯O)], 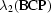 = −292.51 (144.41) exp[−2.90 (29) *d*(H⋯O)] and 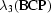 = 233.39 (53.80) exp[−2.09 (13) *d*(H⋯O)]. Dashed lines represent fits to PRIOR values with 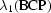 = −261.92 (15.83) exp[−2.87 (4) *d*(H⋯O)], 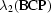 = −200.00 (13.58) exp[−2.76 (4) *d*(H⋯O)] and 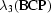 = 176.54 (23.44) exp[−1.92 (7) *d*(H⋯O)]. The dotted line is the function determined by Espinosa, Souhassou, Lachekar & Lecomte (1999 ▶) for multipole densities, with 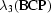 = 410 (80) exp[−2.4 (1) *d*(H⋯O)]. The solid line in (*d*) is the function 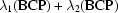 = 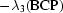.

An exponential dependence on *d*(H⋯O) is also observed for the values of the second derivatives of 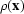 at the BCPs of hydrogen bonds, as they are provided by the three eigenvalues 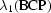, 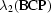 and 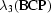 of the Hessian matrix (Fig. 7 ▶). As for the values of the densities themselves, the exponential relationship is almost perfectly fulfilled for the prior, while some scatter of values about the exponential curve can be observed for the eigenvalues derived from the MEM densities. A similar behavior of 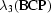 – the curvature at the BCP in the direction of the bond path – has been reported for static multipole densities by Espinosa, Souhassou, Lachekar & Lecomte (1999 ▶). Following the proposal by Espinosa, Souhassou, Lachekar & Lecomte (1999 ▶), 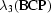 as derived from dynamic MEM densities, might thus form a suitable parameter for the classification of hydrogen bonds.

Values of the Laplacian 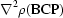 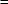 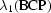 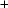 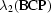 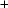 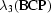 exhibit an exponential dependence on 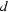
(H⋯O) for the PRIOR, while they show a considerable scatter for the MEM densities (Fig. 6 ▶ *b*). These variations can be explained by the fact that 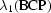 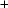 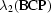 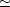 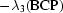 (Fig. 7 ▶ *d*), such that minor variations of the values of the individual eigenvalues are magnified towards large variations of 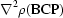. We believe the source of these variations to be, on the one hand, artifacts of the MEM and noise in the data and, on the other hand, variations of the thermal motion between different structures [see the discussion on 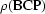 above]. The latter property especially has previously been shown to be an important effect, where relatively small variations of thermal parameters lead to large variations of 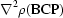, while they hardly effect 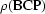 (Hofmann, Netzel & van Smaalen, 2007 ▶).

Because thermal motion depends on the crystal packing, part of the observed variations of 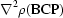 will reflect true variations of the dynamic MEM electron densities, as they are the result from true variations of the thermal motion. Nevertheless, the large scatter and especially the negative values of 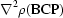 most probably are caused by noise in the data and noise in the MEM density that has an enlarged influence on derivatives due to our method of calculation of derivatives. The outliers of 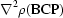 do not belong to a particular dataset, which excludes the explanation that one of the datasets might be particularly affected by noise or systematic errors.

### 3.3. Energetic properties of hydrogen bonds

**Figure 8:**
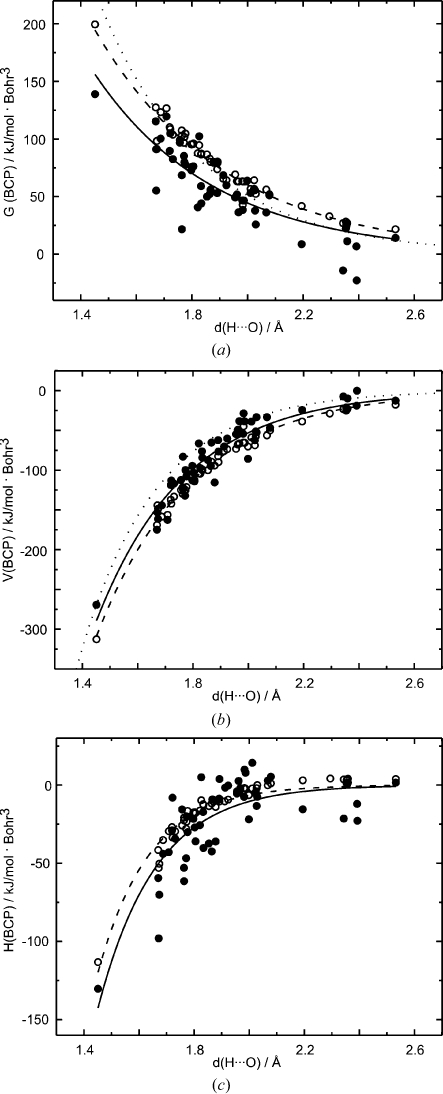
Energetic properties of hydrogen bonds of MEM (filled circles) and prior (open circles) densities depending on the distance *d*(H⋯O). (*a*) Kinetic energy density 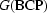, (*b*) potential energy density 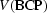 and (*c*) total energy density 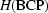. Solid lines represent fits to the MEM values with 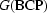 = 4.331 (1.879) × 10^3^ exp[−2.29 (25) *d*(H⋯O)], 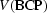 = −2.572 (627) × 10^4^ exp[−3.09 (14) *d*(H⋯O)] and 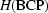 = −1.419 (1.040) × 10^5^ exp[−4.76 (46) *d*(H⋯O)]. Dashed lines correspond to fits to prior values with 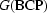 = 4.335 (590) × 10^3^ exp[−2.14 (8) *d*(H⋯O)], 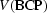 = −2.113 (178) × 10^4^ exp[−2.91 (5) *d*(H⋯O)] and 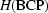 = −2.721 (782) × 10^5^ exp[−5.33 (18) *d*(H⋯O)]. Dotted lines are functions determined by Espinosa *et al.* (1998 ▶) for multipole densities, with 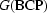 = 12 (2) × 10^3^ exp[−2.73 (9) *d*(H⋯O)] and 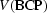 = −50 (1.1) × 10^3^ exp[−3.6 *d*(H⋯O)].

The kinetic, potential and total energy densities at the BCPs of hydrogen bonds have been calculated according to the procedure given in §2 [see (6)–(8) [Disp-formula fd7] [Disp-formula fd8]]. They show a nearly perfect exponential dependence on *d*(H⋯O) for prior densities (Fig. 8 ▶). Corresponding values from MEM densities scatter around an average exponential dependence. A larger or smaller scatter is obtained, depending on the relative importance of 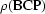 and 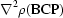 in determining each property [see (6)–(8) [Disp-formula fd7] [Disp-formula fd8]].

Similar exponential relationships have been obtained for static multipole densities by Espinosa *et al.* (1998 ▶). Although the functions for energy densities derived from dynamic MEM densities are different from those for static multipole densities, the differences are smaller than in the case of 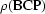 and 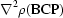. This indicates a compensating effect on going from static to dynamic densities, where, on average, a larger value of 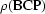 is compensated by a smaller value of 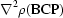 (Fig. 6 ▶). The functionals by Abramov (1997 ▶) describing energy densities at BCPs [see (6)–(8) [Disp-formula fd7] [Disp-formula fd8]] thus give values close to those of static densities, when applied to the dynamic MEM densities described in this article. The restriction to diffraction data measured at very low temperatures (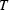 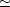 20 K) is an important contribution to the validity of this property of the energy functionals, because these very low temperatures make the thermal motion as small as possible. We therefore believe that the functionals by Abramov (1997 ▶) [see (6)–(8) [Disp-formula fd7] [Disp-formula fd8]] applied to low-temperature, dynamic MEM densities provide a reasonable approximation to the energy densities as they have been defined for the corresponding static densities.

The potential energy density 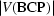 describes the ability of the system to concentrate electrons at the BCPs, while the kinetic energy density 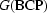 describes the tendency of the electrons to spread out (Espinosa *et al.*, 1998 ▶). Accordingly, values 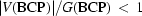 are considered to indicate a depletion of electrons at the BCPs, which corresponds to closed-shell interactions. Values 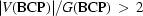 indicate an accumulation of electrons at the BCP, which corresponds to a shared-shell interaction, *i.e.* a covalent bond. Values of 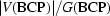 between one and two describe bonds with partial covalent and partial ionic character (Espinosa *et al.*, 2002 ▶, and references therein).

**Figure 9:**
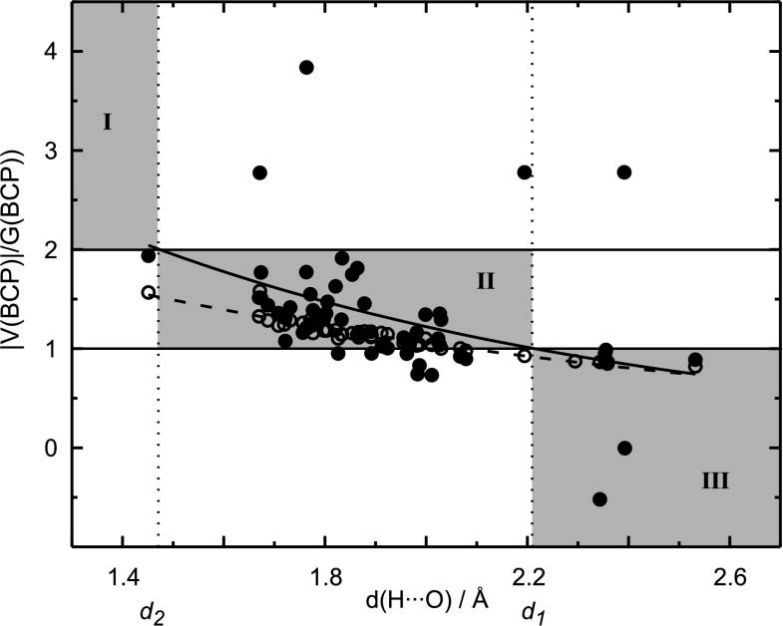
Ratio of potential and kinetic energy densities 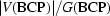 at BCPs of hydrogen bonds of MEM (filled circles) and prior (open circles) densities depending on the distance *d*(H⋯O). The solid line represents the fit to the MEM values with 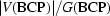 = 7.95 (5.07) exp[−0.94 (34) *d*(H⋯O)]. The dashed line represents the fit to the prior values with 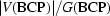 = 4.17 (30) exp[−0.69 (4) *d*(H⋯O)]. 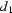 = 2.21 Å and 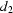 = 1.47 Å.

In agreement with previous studies on multipole densities by Espinosa *et al.* (1998 ▶), we find for hydrogen bonds that both 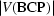 and 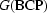 increase on decreasing the distance *d*(H⋯O) (Fig. 8 ▶). However, the relation between 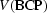 and 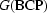 is not linear, such that 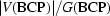 increases with decreasing distance *d*(H⋯O) (Fig. 9 ▶). These relations can again be described by exponential functions. From the average exponential dependence of 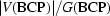 of MEM densities on *d*(H⋯O), two distances can be derived that describe the cross-over between covalent, mixed-character and closed-shell types of hydrogen bonds. The distance 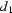 = 2.21 Å is the distance at which 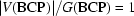 and 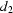 = 1.47 Å is the distance at which 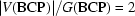. MEM electron densities are thus in agreement with long hydrogen bonds [*d*(H⋯O) 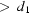] being dominated by electrostatic interactions, while short hydrogen bonds [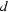
(H⋯O)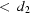] are covalent bonds. Most hydrogen bonds studied in the present work are of intermediate character (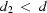
(H⋯O)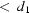 ; see Fig. 9 ▶) and thus at least partly covalent.

The distances 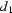 and 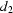 coincide with the classification by Jeffrey (1997 ▶) who considers hydrogen bonds with *d*(H⋯O) > 2.2 Å to be weak and hydrogen bonds with *d*(H⋯O) < 1.5 Å to be strong. MEM electron densities thus indicate that strong hydrogen bonds are covalent bonds, while weak hydrogen bonds possess mainly electrostatic character. Most hydrogen bonds are of intermediate strength and will have mixed covalent–electrostatic character.

A few outliers can be observed in Fig. 9 ▶, for which 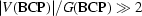 or 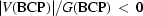. These points are precisely those hydrogen bonds for which a negative Laplacian 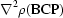 has been obtained (Fig. 6 ▶). Since a negative Laplacian is interpreted as being non-physical for hydrogen bonds, these values of 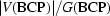 are most probably caused by inaccuracies of the MEM or the data. Deviations from a smooth dependence on the distance might also arise from the approximate character of (6)–(8) [Disp-formula fd7] [Disp-formula fd8] (Abramov, 1997 ▶), and from the fact that these relations have not been derived for dynamic densities.

### 3.4. Topological and energetic properties of covalent bonds

**Figure 10:**
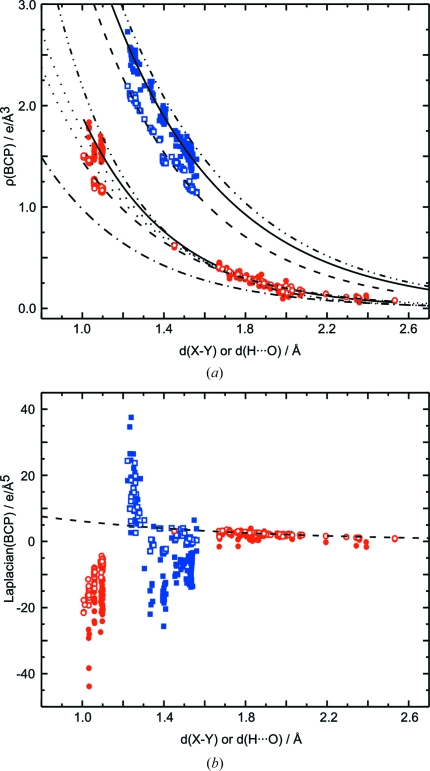
Topological properties at BCPs from MEM (filled symbols) and prior (open symbols) densities depending on the bond length *d*(*X*—*Y*) or distance *d*(H⋯O). Covalent bonds C—O, C—N, C—C (blue squares) are distinguished from covalent C—H, covalent N—H and hydrogen bonds (red circles). (*a*) 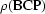 and (*b*) 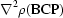. Solid lines represent fits to MEM values with 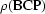 = 23.21 (95) exp[−1.79 (3) *d*(H⋯O)] for C—O, C—N and C—C bonds and 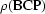 = 18.45 (99) exp[−2.28 (5) *d*(H⋯O)] for C—H, N—H and hydrogen bonds. Dashed lines correspond to fits to prior values with 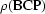 = 23.47 (98) exp[−1.95 (3) *d*(H⋯O)] for C—O, C—N and C—C bonds and 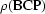 = 10.71 (35) exp[−2.00 (3) *d*(H⋯O)] for C—H, N—H and hydrogen bonds. Dotted lines represent the fits to values for hydrogen bonds from Fig. 6 ▶(*a*). The dash–dotted line is the function determined by Espinosa, Souhassou, Lachekar & Lecomte (1999 ▶) from multipole values for hydrogen bonds (Fig. 6 ▶ *a*). Dash–dot–dotted lines are functions from Dominiak *et al.* (2006 ▶) with 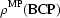 = exp[−1.74 (4) (*d*(*X*—*Y*) − 1.822 (10))] for C—O, C—N and C—C bonds, and with 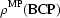 = exp[−2.61 (5) (*d*(*X*—*Y*) − 1.300 (4))] for covalent C—H, N—H and O—H bonds and hydrogen bonds.

Densities at the BCPs of covalent bonds are found to depend exponentially on the bond length *d*(*X*—*Y*). The bonds C—O, C—N and C—C require a different function than the covalent bonds C—H and N—H and hydrogen bonds, while all bonds involving H atoms are described by a single curve (Fig. 10 ▶ *a*). A more detailed analysis of the values 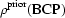 shows that slightly different exponential curves apply to values obtained from different bond types C—O, C—N and C—C, in agreement with the behavior of 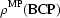 obtained from multipole densities by Dominiak *et al.* (2006 ▶). A similar analysis cannot be made for 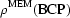, because of the relatively few data points for each bond type and the larger scatter about the average exponential relation.

The exponential dependencies of 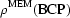 and 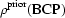 on *d*(H⋯O) as determined from hydrogen bonds [1.4 < *d*(H⋯O) < 2.6 Å; Fig. 10 ▶ *a* and the dotted lines in Fig. 10 ▶ *a*] extrapolate well towards values of 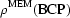 and 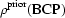 for covalent bonds C—H and N—H [1.0 < *d*(H⋯O) < 1.2 Å; Fig. 10 ▶ *a*]. This remarkable feature indicates that a different trend exists in the values 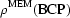 and 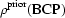 of hydrogen bonds, despite the fact that their magnitudes are within the same range and that the fitted curves hardly differ within the hydrogen-bond region (Fig. 6 ▶ *a*). Fig. 10 ▶(*a*) shows that the similar magnitudes of 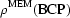 and 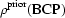 of hydrogen bonds are a coincidental feature, because the different trends show that – for purposes of characterizing hydrogen bonds – the true density cannot be replaced by the procrystal density, as it has sometimes been suggested (Spackman, 1999 ▶; Downs *et al.*, 2002 ▶).

Previously we have shown that for most bonds in trialanine and α-glycine 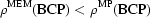, while the opposite is true for hydrogen bonds (Hofmann, Netzel & van Smaalen, 2007 ▶; Netzel *et al.*, 2008 ▶). The present analysis shows that, on average, this property is valid for all covalent bonds of the six compounds studied. The exponential dependence of 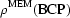 of H—*X* covalent and H⋯O hydrogen bonds intersects the function determined for 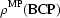 by Dominiak *et al.* (2006 ▶) at a distance of 1.44 Å, such that 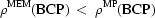 when 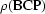 is large and 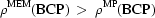 when 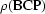 is small. Fig. 10 ▶(*a*) suggests that a similar property would be valid for the van der Waals contacts, but an extrapolation towards large distances [*d*(*X*—*Y*) > 3 Å] does not seem permissable for the given accuracy and range of data points.

**Figure 11:**
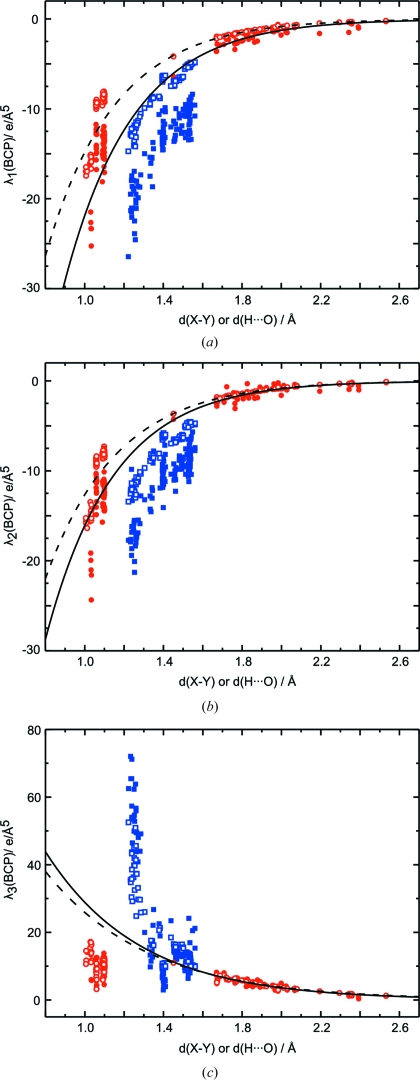
Curvatures at BCPs from MEM (filled symbols) and prior (open symbols) densities depending on the bond length *d*(*X*—*Y*) or distance *d*(H⋯O). Covalent bonds C—O, C—N, C—C (blue squares) are distinguished from covalent C—H and N—H and hydrogen bonds (red circles). (*a*) 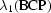, (*b*) 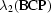 and (*c*) 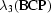. Lines represent fits to the values for hydrogen bonds from MEM (solid lines) and prior (dashed lines) densities, as taken from Fig. 7 ▶.

Eigenvalues of the Hessian matrix at the BCPs show a systematic variation with the bond length, which can be described by an exponential function within the limited range of distances *d*(H⋯O) of hydrogen bonds (Figs. 7 ▶ and 11 ▶). While each type of bond seems to require its own curve, the limited number of data points does not allow these functions to be determined. Unlike 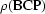, the values of distance dependencies of 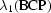, 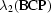 and 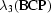 of hydrogen bonds do not extrapolate well towards corresponding values for covalent N—H and C—H bonds. Instead, each type of covalent bond exhibits a large variation of values of the second derivatives, while being of almost a single length (Fig. 11 ▶). Not so dramatic, the distance dependencies exhibit similar features for the values of the second derivatives at the BCPs of other covalent bonds. This property is enhanced for the values of 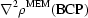 and 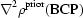 (Fig. 10 ▶ *b*). While a systematic dependence of 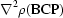 on the bond distance has been reported for values derived from multipole densities (Dominiak *et al.*, 2006 ▶), a close inspection of the published diagrams shows that 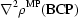 exhibits similar features as presently found for 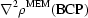 and 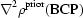, *i.e.* different values for bonds of nearly equal length.

**Figure 12:**
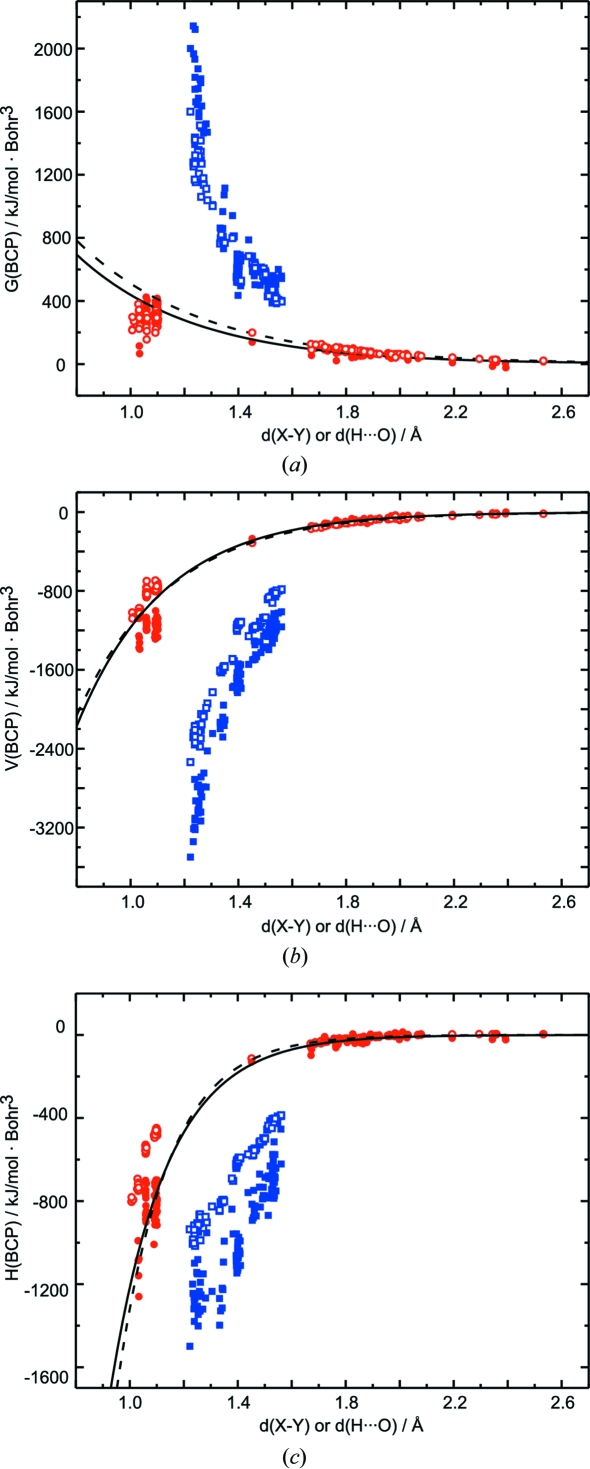
Energetic properties at BCPs from MEM (filled symbols) and prior (open symbols) densities depending on the bond length *d*(*X*—*Y*) or distance *d*(H⋯O). Covalent bonds C—O, C—N, C—C (blue squares) are distinguished from covalent C—H and N—H, and hydrogen bonds (red circles). (*a*) Kinetic energy density 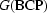, (*b*) potential energy density 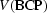 and (*c*) total energy density 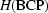. Lines represent fits to the values for hydrogen bonds from MEM (solid lines) and prior (dashed lines) densities, as taken from Fig. 8 ▶.

**Figure 13:**
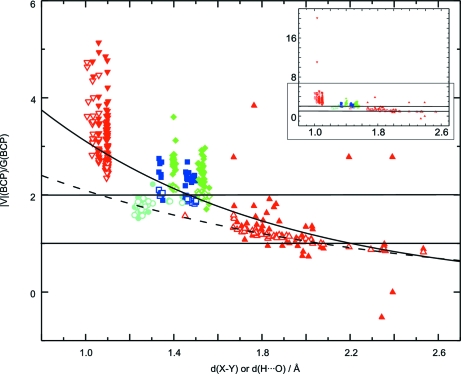
The ratio 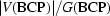 from MEM (filled symbols) and prior (open symbols) densities depending on the bond length *d*(*X*—*Y*) or distance *d*(H⋯O). Indicated are values for C—O (light green circles), C—N (blue squares), C—C (green diamonds), C—H (upside-down triangles) and N—H (red upside-down triangles) covalent bonds, and for hydrogen bonds (red triangles). The solid line (MEM values) and the dashed line (prior values) represent fits to values for hydrogen bonds from Fig. 9 ▶. The inset shows all values of 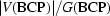, including two very large ratios for N—H bonds.

The properties of the distance dependencies of 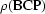 and 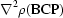 are transported towards the energy densities at the BCPs (Fig. 12 ▶). They are even more pronounced for the values of 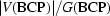. 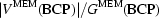 assumes a large range of values for covalent bonds C—C, C—N, C—H and N—H, while distances of these bonds cluster around a few values only (Fig. 13 ▶). This suggests a variation in the character of bonds of similar length, as might be the result of different environments of these bonds.

Polar C—O bonds appear to be of mixed covalent/ionic character with 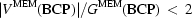, while bonds C—C, C—N, C—H and N—H appear to be covalent with 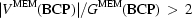 (Fig. 13 ▶), in accordance with general chemical knowledge. For covalent bonds C—C and C—N 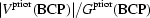 is close to two and generally much smaller than 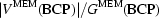. This again shows that the true density should not be replaced by the procrystal density for the quantitative description of chemical bonding.

## 4. Conclusions

Charge densities have been determined by the MEM from X-ray diffraction data on six different crystals of amino acids and tripeptides. Employing the previously proposed criterion of convergence for the iterations of the MEM (Hofmann, Netzel & van Smaalen, 2007 ▶), the values of 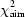 have been found to vary by a factor of four. These values correlate with the GoF of the multipole refinements (Table 1 ▶), and they thus show the ability of the MEM to determine the correct scale of standard uncertainties of measured intensities (§2.1).

Electron densities 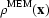 and 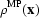 exhibit similar features, with atomic maxima and BCPs at similar positions (Hofmann, Netzel & van Smaalen, 2007 ▶; Netzel *et al.*, 2008 ▶). Differences are due to the differences between dynamic [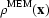] and static [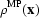] densities as well as the peculiarities of each method. Electron densities in hydrogen bonds have been found to be better represented by the MEM than by multipole models (§3.1), as it is the result of the inflexibility of the multipole model for the small number of poles (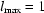) that has been used for H atoms (Volkov *et al.*, 2000 ▶, 2001 ▶; Volkov & Coppens, 2001 ▶; Madsen *et al.*, 2004 ▶; Koritsanszky, 2006 ▶).

MEM densities at BCPs show an exponential dependence on the bond length with individual functions for covalent bonds between non-H atoms and bonds involving H atoms. These functions differ from the functions that have been determined for multipole densities at BCPs (Figs. 6 ▶ *a* and 10 ▶ *a*) (Espinosa, Souhassou, Lachekar & Lecomte, 1999 ▶; Dominiak *et al.*, 2006 ▶). In general, 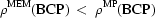 for covalent bonds, while the opposite is true for hydrogen bonds (§3.4).

Values of 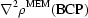 exhibit a larger scatter about exponential dependencies on bond lengths than the corresponding values from multipole densities. Nevertheless, it proved possible to establish systematic dependencies of energetic properties at BCPs of MEM densities on the bond length (Fig. 12 ▶). In particular, the ratio between potential and kinetic energy density shows two kinds of behavior.

For hydrogen bonds, 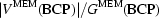 allows a classification of hydrogen bonds according to their distance *d*(H⋯O) (§3.3). Short hydrogen bonds [*d*(H⋯O)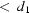 = 1.47 Å] are covalent bonds, hydrogen bonds of intermediate length [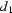 < *d*(H⋯O) < 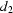 = 2.21 Å] possess mixed covalent–ionic character, while long hydrogen bonds [*d*(H⋯O)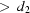] are mainly stabilized by closed-shell-type interactions. This classification coincides with the usual classification of strong [*d*(H⋯O) < 1.5 Å], intermediate [1.5 < *d*(H⋯O) < 2.2 Å], and weak [*d*(H⋯O) > 2.2 Å] hydrogen bonds (Jeffrey, 1997 ▶).

For covalent bonds, the ratio 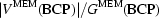 assumes values within a large range for each type of bond with a narrow range of bond lengths (Fig. 13 ▶). This feature indicates that the character of covalent bonds of a single type [*e.g.* C(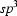)—C(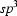) bonds] varies despite almost equal bond lengths. A classification of bonds according to their length can therefore capture at most part of the chemistry.

The procrystal prior is only rarely considered in charge-density studies (Downs *et al.*, 2002 ▶). Here it has been shown that topological properties at BCPs exhibit similar dependencies on bond lengths when derived from MEM and procrystal densities, while the latter values show much less scatter. In particular, most of the density in the BCP is already described by the procrystal density (Fig. 10 ▶ *a*), which illustrates the difficulties of MEM calculations and multipole refinements in establishing a charge density beyond the procrystal model. Differences between MEM and procrystal densities are more pronounced in the energy densities. This suggests that only the true charge densities – whether obtained by the MEM, the multipole model or some other method – may lead to a correct interpretation of the character of bonds.

Different trends could be identified in the distance dependencies of 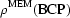 and 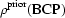 of hydrogen bonds, despite almost equal values of 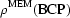 and 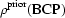 for these bonds (Figs. 6 ▶ *a* and 10 ▶ *a*). This remarkable feature stresses that MEM and procrystal densities are different, and it shows once more that – for purposes of characterizing chemical bonding in hydrogen bonds – the true density cannot be replaced by the procrystal density, as has sometimes been suggested (Spackman, 1999 ▶; Downs *et al.*, 2002 ▶).

## Figures and Tables

**Table 1:** Crystallographic data of α-glycine (Gly; Destro *et al.*, 2000 ▶), L-alanine (Ala; Destro *et al.*, 1988 ▶), L-phenylalanine formic acid complex (Phe; Mebs *et al.*, 2006 ▶), trialanine (Ala–Ala–Ala; Rödel *et al.*, 2006 ▶), L-alanyl-L-tyrosyl-L-alanine with water [Ala–Tyr–Ala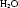; Checinska *et al.*, 2006 ▶], and L-alanyl-L-tyrosyl-L-alanine with ethanol [Ala–Tyr–Ala_(EtOH)_; Checinska *et al.*, 2006 ▶], together with summaries of the ISAM refinements and MEM calculations (present work) Reflections with 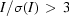 are classified as observed, with the exception of the criterion 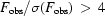 for trialanine (Rödel *et al.*, 2006 ▶).

Compound	Gly	Ala	Phe	Ala–Ala–Ala	Ala–Tyr–Ala_(H_2_O)_	Ala-Tyr-Ala_(EtOH)_
Chemical formula	C 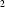 O 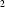 NH 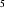	C 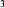 O 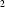 NH 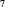	C 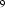 H 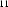 NO 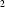 ·C 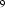 H 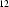 NO 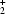 ·CHO 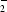	C 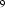 H 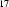 N 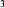 O 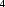 ·H 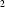 O	C 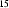 H 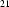 N 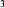 O 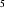 ·2.634H 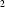 O	C 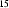 H 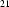 N 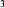 O 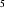 ·C 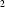 H 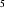 OH
Space group	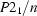	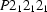	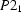	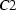	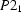	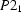
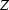	4	4	2	8	2	2
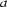 (Å)	5.0866	5.9279	11.4585	18.4408	8.121	8.845
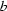 (Å)	11.7731	12.2597	5.5941	5.2153	9.299	9.057
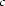 (Å)	5.4595	5.7939	14.2147	24.8543	12.532	12.364
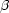 (°)	111.99	90.00	99.46	98.76	91.21	94.56
*V* (Å 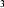 )	303.16	421.1	898.8	2362.4	946.2	987.3
*F*(000)	160	192	400	1031.8	397	396
Temperature (K)	23	23	25	20	9	20
Wavelength 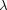 (Å)	0.71073	0.71073	0.71073	0.71073	0.50000	0.71073
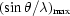 (Å 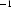 )	1.15	1.08	1.18	1.15	1.24	1.11
Unique reflections (obs/all)	3483/3822	2328/2535	8971/10981	12928/14895	12875/14111	10901/11703
						
**Multipole refinement** (Destro *et al.*, 1988 ▶, 2000 ▶; Mebs *et al.*, 2006 ▶; Checinska *et al.*, 2006 ▶; Rödel *et al.*, 2006 ▶)						
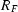 (obs/all)	–/0.0129	–/0.0203	0.0272/0.0350	0.0183/0.0247	0.0293/0.0351	0.0223/0.0264
*R* 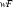 (obs/all)	–/–	–/0.0159	0.0307/–	0.0153/–	0.0208/–	0.0177/–
GoF	1.04	1.17	1.06	0.67	2.06	1.63
						
**ISAM refinement**						
*R* 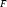 (obs/all)	0.0233/0.0260	0.0285/0.0316	0.0404/0.0480	–	0.0399/0.0455	0.0360/0.0400
*R* 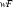 (obs/all)	0.0525/0.0535	0.0373/0.0377	0.0473/0.0487	–/–	0.0478/0.0494	0.0419/0.0425
GoF(obs/all)	2.02/1.96	1.81/1.75	1.43/1.33	–/–	1.67/1.64	2.27/2.22
						
**MEM calculation** (this work; Netzel *et al.*, 2008 ▶; Hofmann, Netzel & van Smaalen, 2007 ▶)						
Number of pixels	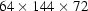	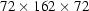	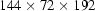	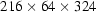	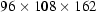	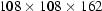
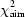	0.3131	0.7600	0.8300	0.4250	1.2750	1.3081
*R* 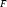 /*R* 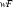	0.0104/0.0153	0.0199/0.0190	0.0355/0.0343	0.0263/0.0184	0.0330/0.0342	0.0248/0.0222
